# Parakinesia Brachialis Oscitans – a Rare Post-Stroke Phenomenon

**DOI:** 10.5334/tohm.680

**Published:** 2022-03-11

**Authors:** Abhishek Chowdhury, Amlan Kusum Datta, Samar Biswas, Atanu Biswas

**Affiliations:** 1 Department of Neurology, Institute of Post Graduate Medical Education & Research and Bangur Institute of Neuroscience, Kolkata, India

**Keywords:** involuntary movement, ischemic stroke, parakinesia brachialis oscitans, paralysis, yawning

## Abstract

**Background:**

Abnormal involuntary movement of paralyzed upper limb during yawning is a rare phenomenon termed as parakinesia brachialis oscitans.

**Case Report:**

We describe a 59-year-old gentleman with abnormal involuntary movement of paralyzed right upper limb during yawning 2 weeks following ischemic stroke of left middle cerebral artery territory.

**Discussion:**

This is a rare post-stroke phenomenon and its pathophysiological mechanism is poorly understood but this entity highlights possible preserved extrapyramidal pathway which might help in rehabilitating stroke survivors.

## Introduction

The rare phenomenon of abnormal involuntary movement of paralyzed upper limb in association with yawning following stroke was termed as parakinesia brachialis oscitans (PBO) by Walusinski et al. [1]. Only few cases have been described in the literature and there are many unanswered question of this clinical condition. We report a patient of PBO after acute infarction in left middle cerebral artery (MCA) territory.

## Case Report

A 59-year-old right-handed gentleman presented in the Neurology outpatient department (OPD) with history of acute onset right hemiparesis and motor aphasia of 4-weeks duration. He was a known diabetic, hypertensive and smoked 1 packet of cigarettes per day for 30 years. There was no past or family history of stroke or myocardial infarction. His CT scan of brain did not reveal any intra-parenchymal haemorrhage or any evidence of infarct. He was treated by his physician soon after the stroke and was gradually improving.

About 2 weeks following the stroke, his relatives noticed an involuntary lifting of the hemiplegic right arm with yawning. On observing the patient at OPD, this abnormal movement was confirmed. He was still having weakness of his right sided limbs (***Video 1***). The movement was synchronous with every episode of yawning. The movement consisted of slow progressive abduction of shoulder, flexion and mild supination at elbow, associated with occasional dorsiflexion of wrist. The phenomenon was observed with each episode of yawning (***Video 2***). It was of same duration as the episode of yawning. It started shortly after the start of yawn with the hand getting lifted up during the entire inhalation phase of the yawn and then quickly returned to its original position during the brief exhalation phase. The patient remained fully conscious throughout the movement. There were no other associated movements noticed in his face, left upper limb or lower limbs.

**Video 1:** **Hemiplegia.** Shows weakness of right upper limb.

**Video 2:** **PBO.** Shows involuntary movement of right upper limb during yawning.

Besides motor aphasia there was spasticity of his right upper and lower limb and normal tone in his left side. There was right sided upper motor neuron facial weakness and right hemiparesis with power of grade 2/5 in upper limb and 3/5 in lower limb (Medical Research Council) and normal power on left side. There were exaggerated tendon reflexes and positive Babinski sign on right side and equivocal plantar response on the left.

**Figure 1:**
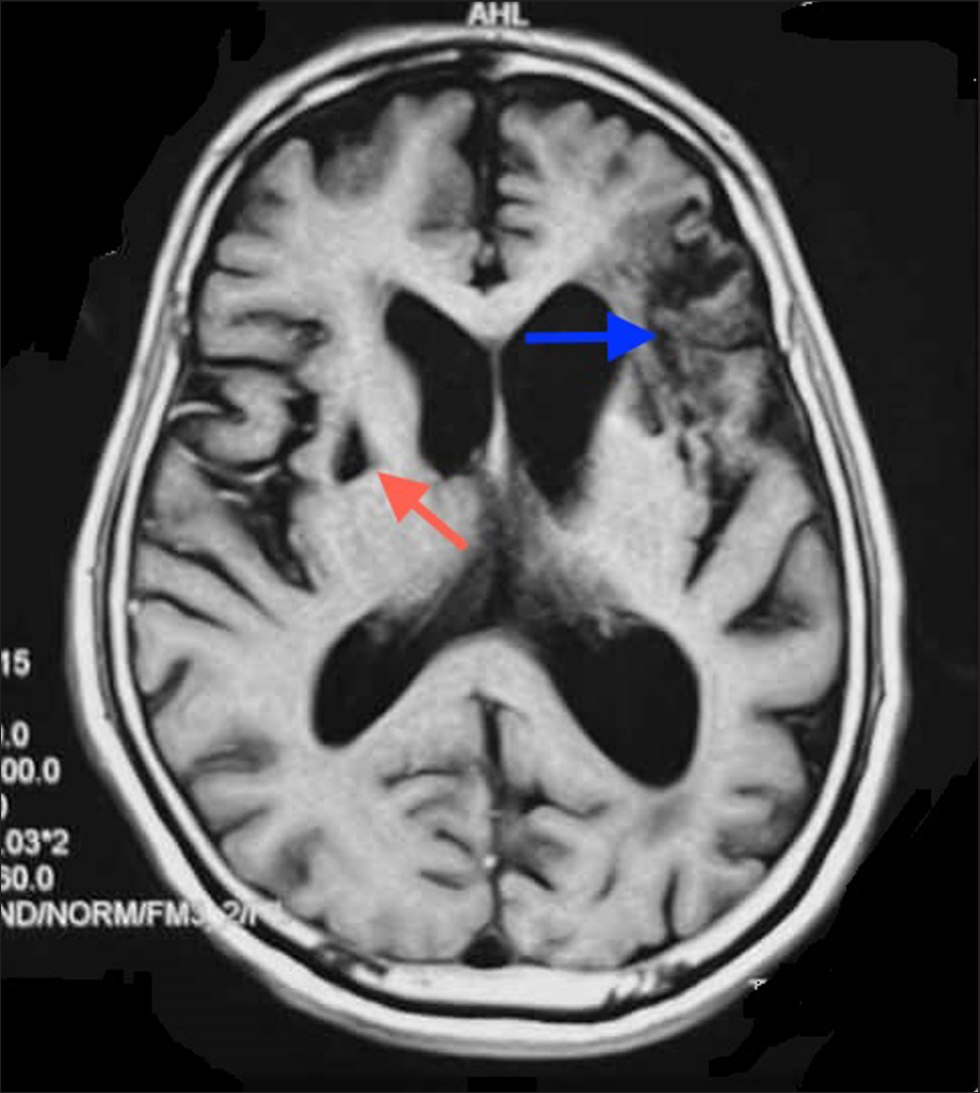
Axial T1 weighted MR image shows irregular signal characteristics in left lateral frontal cortex and white matter extending to operculum, precentral gyrus, sylvian cortex (blue arrow) as well as gliosis in right putamen and head of caudate nucleus (red arrow).

**Figure 2:**
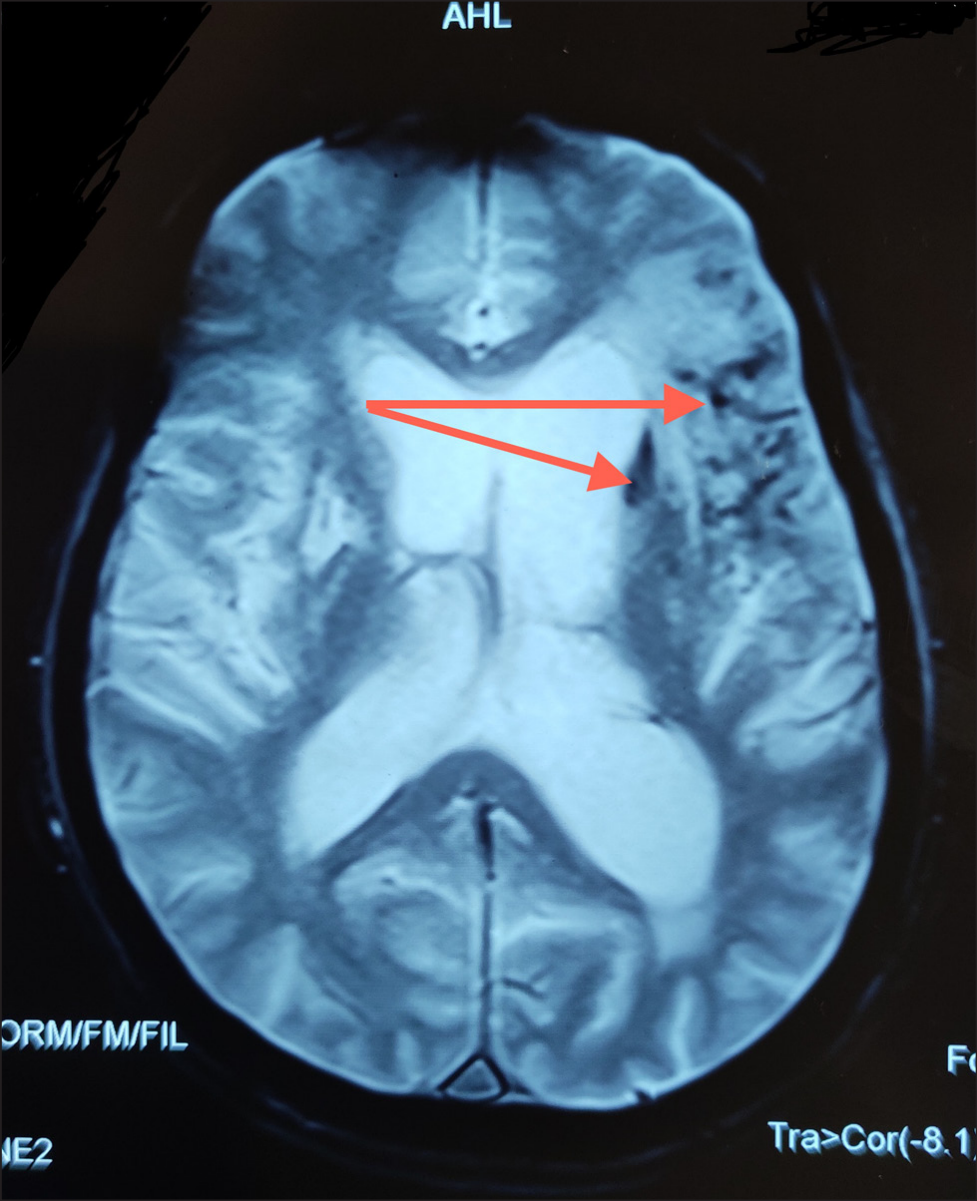
Axial Gradient Echo Image shows internal hypointense foci with blooming effect suggesting haemorrhage (red arrows).

**Figure 3:**
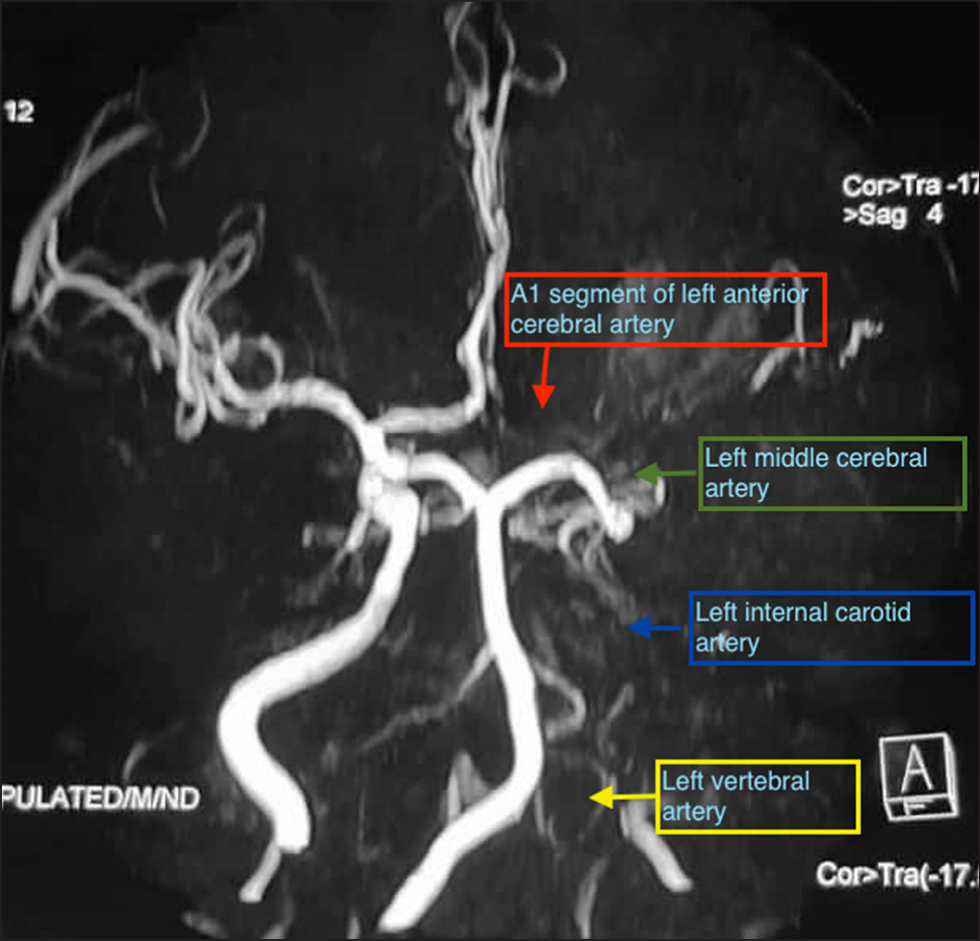
MR angiogram shows loss of flow signal in intracranial left internal carotid artery (blue arrow), left middle cerebral artery (green arrow) and A1 segment of left anterior cerebral artery (red arrow). Left vertebral artery (yellow arrow) remains non visualized.

MRI brain showed a chronic infarction in left lateral frontal cortex and white matter extending to operculum, precentral gyrus, sylvian cortex and basal ganglia with evidence of haemorrhagic transformation (***Figures 1 and 2***). MR angiography revealed loss of flow signal in intracranial left internal carotid artery and left MCA (***Figure 3***). A1 segment of left anterior cerebral artery was hypoplastic with patent anterior communicating artery. Doppler ultrasound of bilateral carotid and vertebral arteries showed atherosclerotic plaques in bilateral carotid arteries without significant occlusion. Electrocardiogram and echocardiogram were suggestive of left ventricular hypertrophy without any evidence of atrial fibrillation, intracavitary thrombus or any valvular pathology.

He was managed conservatively with antiplatelets, high dose statins, antihypertensives, oral hypoglycaemic agents, physiotherapy and speech therapy. Since then the patient is on regular follow up in Neurology OPD. There has been significant improvement of his motor power and speech functions in the last one year. The abnormal movements are still persisting, however there has been a decrease in the degree of lifting of the paretic limb.

## Discussion

Cases of movement of paralysed arm in hemiplegic limbs which were completely disobedient to the will was known for a long. However, Walusinski et al. in 2010 proposed the term parakinesia brachialis oscitans (PBO) for this rare phenomenon [1]. The term parakinesia, as defined by the authors, means “an abnormal involuntary movement that acts as a parasite, caricature or replacement of a normal movement” and oscitans means “yawn” in Latin [1].

This movement is typically seen in the paralysed upper limb in synchrony with the yawning episode. However, two cases were reported to have some associated movements in the hemiplegic lower limb [2] and may be considered as variants of PBO. These involuntary, patterned movements usually include abduction and internal rotation of shoulder, flexion of elbow and extension of fingers. These are usually slowly progressive movements which start with the onset of yawn and continues throughout the inhalation phase of the yawn followed by a swift return of the affected limb to its original position.

In one of the reported cases [3], there was tremor associated with the movement. In another reported case, patient could perform complex purposeful act during movement and also could wilfully supress it [4]. It usually takes about 5 seconds for the entire movement. In most of the cases, these are observed soon after the acute insult (generally within first week), however late onset during the spastic phase may also be seen. There was no suggestion of any age or gender predilection but most reported cases of PBO were men. Also, there was no preference for any laterality or dominance. These movements tend to disappear with recovery of motor function in the affected limb, usually within six months. However, it may persist for longer periods as seen in our patient. The most common etiologies which were found in reported cases of PBO were stroke. Although both ischemic and haemorrhagic strokes were found to be associated with PBO, most of the cases available in literature are ischemic stroke. Meenakshisundaram et al. (2010) studied 75 patients with abnormal movement during yawning following acute stroke and reported a mean onset time of 36 to 38 hours in male and females, respectively [5]. Associated movements in hemiplegic limbs during yawning were minimal and observed in 78.6%, which was significantly more in males (83% vs 70%), in those with hypotonia (87% vs 61%)), and in proximal joints (72% vs 29%) irrespective of limb. It was also more common in left-sided (94% vs 64%) hemiplegics, and in the upper limbs (91% vs 83%). ***Table 1*** shows cases of PBO reported by researchers from various countries.

**Table 1:** Comparison of cases of parakinesia brachialis oscitans (PBO) described in literature.


SR NO.	REFERENCES	AGE/SEX	SITE OF LESION	INFARCT/HAEMORRHAGE	PBO ONSET	DURATION OF PBO	MOVEMENT DESCRIPTION

*1*.	*Blin et al. (France -1993)* [6]	62/M	Posterior limb of left internal capsule	Infarct	Few days from stroke onset	>6 weeks	Abduction, antero-flexion and internal rotation of shoulder with mild flexion of elbow and extension of fingers

*2*.	*Topper et al. (Germany- 2002)* [7]	62/M	Total Right MCA territory	Infarct		NA	Spontaneous movements of left arm during yawning

		51/M	Left thalamus and posterior portion on internal capsule	Haemorrhage	After 2 weeks from stroke onset	NA	Right shoulder abduction with extension of right forearm and fingers

		43/M	Right sided pons extending to cerebellar peduncle	Infarct	After 4 days from stroke onset	NA	Abduction and extension of left arm

*3*.	*Walusinski et al. (France – 2010)* [1]	49/F	Posterior limb of right internal capsule	Infarct	After 2 days from stroke onset	2 weeks	Hemiplegic arm rising up to the level of chest

		73/M	Anterior limb of left internal capsule	Infarct	NA	1 week	Hemiplegic arm 30 cms upwards moving

		71/M	Right centrum semiovale and lenticular nucleus	Infarct	From stroke onset	>3 years	Rising of left arm with adduction and elbow flexion

		53/M	Right centrum semiovale and caudate nucleus	Infarct	From stroke onset	>1 year	Rising of left arm with adduction and elbow flexion

		75/M	Left internal capsule and lenticular nucleus	Infarct	From stroke onset	NA	Rising of right arm with adduction and elbow flexion

		35/F	Total left MCA territory	Infarct	From stroke onset	NA	Rising of right arm elbow Flexion

*4*.	*Jung et al. (Republic of korea – 2011)* [8]	59/M	Right precentral gyrus and frontal subcortex	Infarct	Day 2	NA	Left arm rising with adduction and elbow flexion

*5*.	*de Lima PM et al. (Brazil- 2011)* [2]	63/M	Right cortical/subcortical fronto-parietal region	Infarct	After months 2	NA	Abduction and elevation of left arm with minimal extension of forearm

		39/M	Base of right pons	Infarct	From stroke onset	Approx 4 months	Rising of left arm (abduction and flexion) with extension of lower limb

		55/M	Right rostral medulla	Infarct	From Stroke onset	NA	Left Upper and Lower limb presented with contractures while yawning

*6*.	*Zorzetto et al. (Brazil – 2013)* [9]	60/M	Left MCA territory	Infarct	From stroke onset	24 hours	Rising of right arm with adduction and elbow flexion

*7*.	*Yung-Tsan Wu et al. (Taiwan – 2013)* [10]	52/M	Right putamen	Haemorrhage	4 months after stroke	NA	Abduction and mild internal rotation of shoulder with elbow flexion

*8*.	*Farah et al. (Canada – 2015)* [3]	51/M	Left fronto-parieto-temporal region	Infarct	After 2 days	12 hours	Rising of right arm associated with tremor

*9*.	*Kang P, Dhand A (USA – 2015)* [12]	63/<	Left MCA territory involving cortical and subcortical structures	Infarct	From stroke onset	NA	With yawning, right arm consistently rose to chest, movement ceased after 2 weeks following partial recovery of arm strength (MRC grade 4)

*10*.	*Alves PN et al. (Portugal – 2017)* [4]	59/M	Left MCA territory involving the anterior limb of internal capsule, and of the anterior, posterior, and inferior regions of putamen	Infarct	From stroke onset		Reflexive, stereotyped movement of flexion of the right elbow while yawing. On day 6, the patient started to have voluntary control over that movement, during more sustained yawning, he could even perform more complex movements of distal joints, e.g. grabbing objects purposely. These movements could also be volitionally suppressed.

*11*.	*Aaron et al. (Oman – 2019)* [11]	53/M	Right pons and upper medulla	Infarct	After 2 days	3 days	Rising of left arm with yawning

*12*.	*Present case (India – 2021)*	59/M	Left frontal cortex and basal ganglia	Infarct	After 2 weeks	>1 year	Abduction of shoulder, flexion and mild supination at elbow and intermittently associated with dorsiflexion of wrist


The two common sites of lesion were internal capsule and pontomedullary region. Other sites include centrum semiovale, frontal subcortex and total MCA territory infarct.

Yawning is a physiological phenomenon seen in almost all vertebrates. The paraventricular nucleus of hypothalamus plays the key role in yawning through its connections with hippocampus and brain stem areas such as reticular formation and locus coeruleus [3]. The final executive pathway of yawning involves motor nuclei of cranial nerves V, VII, IX, XI and XII and C1–C4. Neocortical brain areas possibly exert an inhibitory effect on these subcortical structures.

The pathophysiology of PBO is still not clear. Walusinski et al. proposed that in PBO there is interruption of corticospinal, corticobulbar and corticopontocerebellar pathways [1]. However, the proprioceptive loop carrying signals between the paleocerebellum, lateral reticular nucleus and motor anterior spinal horn remains functional. So during yawning, strong contractions of respiratory muscles lead to generation of proprioceptive signals that reaches the lateral reticular nucleus. Motor signals from lateral reticular nucleus then travel via extrapyramidal pathways to the anterior horn cells of C4–C8 causing movement of the affected upper limb.

Interruption of cortico-pontocerebellar pathway is essential for PBO so that proprioceptive loop remains disinhibited. This interruption can occur at two levels, at internal capsule affecting the first order neurons or at the level of pons affecting the second order neurons, the two most common sites of lesion for PBO.

Other suggested pathophysiologic mechanism for PBO include disinhibition of subcortical structures by cortical damage that may release the reticular brainstem formation interconnected with motor pathways and may be activated by stimuli such as yawning [6]. Other explanation includes that an “emotional motor system” may be responsible for the movement of paralysed upper limb and yawning would activate it as a consequence of emotional state related to drowsiness [7].

Our patient developed PBO two weeks after a large haemorrhagic infarct in the left lateral frontal cortex and white matter extending to operculum, precentral gyrus, sylvian cortex and basal ganglia interrupting the descending pyramidal tracts. The preserved proprioceptive loop in presence of interrupted corticospinal, corticobulbar and corticopontocerebellar pathways seems to be the most likely explanation for PBO in our patient. Although there was a gliosis suggesting old infarct in the opposite hemisphere, it is difficult to associate that with the phenomenon.

## Conclusion

PBO is a rare and interesting phenomenon seen commonly in post-stroke patients. The exact pathophysiological mechanism is still unknown but it generates tremendous curiosity. Understanding this complex phenomenon might help in rehabilitation of stroke survivors by manipulating preserved extrapyramidal pathways.
